# Supplementary material for: Large-scale capsid-mediated mobilisation of bacterial genomic DNA in the gut microbiome
Source: Nat Commun. 2026 Jan 27;17:2046. doi: 10.1038/s41467-026-68726-4 (PMC12946183; doi:10.1038/s41467-026-68726-4)
Supplement: Supplementary file 1 — Supplementary Information [file 41467_2026_68726_MOESM1_ESM.pdf]

Figure S1

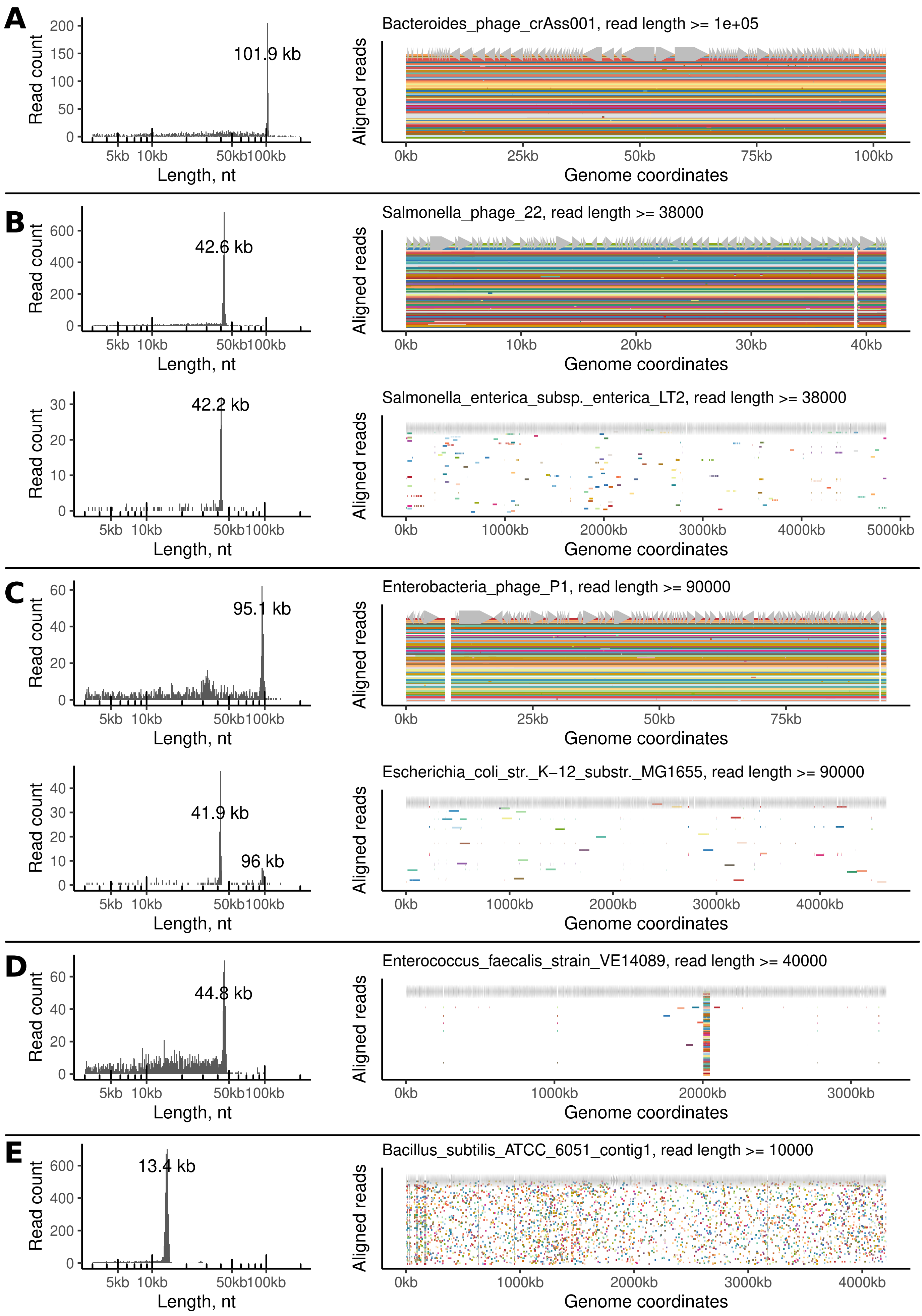
A
Read count
101.9 kb
Length, nt
Bacteroides_phage_crAss001, read length >= 1e+05
Aligned reads
Genome coordinates
B
42.6 kb
Salmonella_phage_22, read length >= 38000
42.2 kb
Salmonella_enterica_subsp._enterica_LT2, read length >= 38000
C
95.1 kb
Enterobacteria_phage_P1, read length >= 90000
41.9 kb
96 kb
Escherichia_coli_str._K−12_substr._MG1655, read length >= 90000
D
44.8 kb
Enterococcus_faecalis_strain_VE14089, read length >= 40000
E
13.4 kb
Bacillus_subtilis_ATCC_6051_contig1, read length >= 10000

Figure S1. Size distribution and mapping of full-sized nanopore VLP DNA reads obtained from known transducing/packaging phage-bacterium systems. A, *Bacteroides* phage crAss001. No reads were detected aligning to the genome of the host strain *Bacteroides intestinalis* APC919/174; **B,** *Salmonella* phage P22 infecting its host *S. enterica* LT2, with reads aligning to both the phage and the host genomes (GT); **C,** Enterobacteria phage P1 infecting its host *E. coli* MG1655 (GT); **D,** induction of prophage pp5 in *E. faecalis* VPE14089, ~44.8 kb-long reads aligning to the prophage region and the adjacent chromosomal region primarily to one side of the prophage, in line with the expected LT pattern[12]; **E,** *B. subtilis* GTA-like element PBSX, ~13.4 kb-long reads show uniform coverage of the entire *B. subtilis* genome.

Figure S2

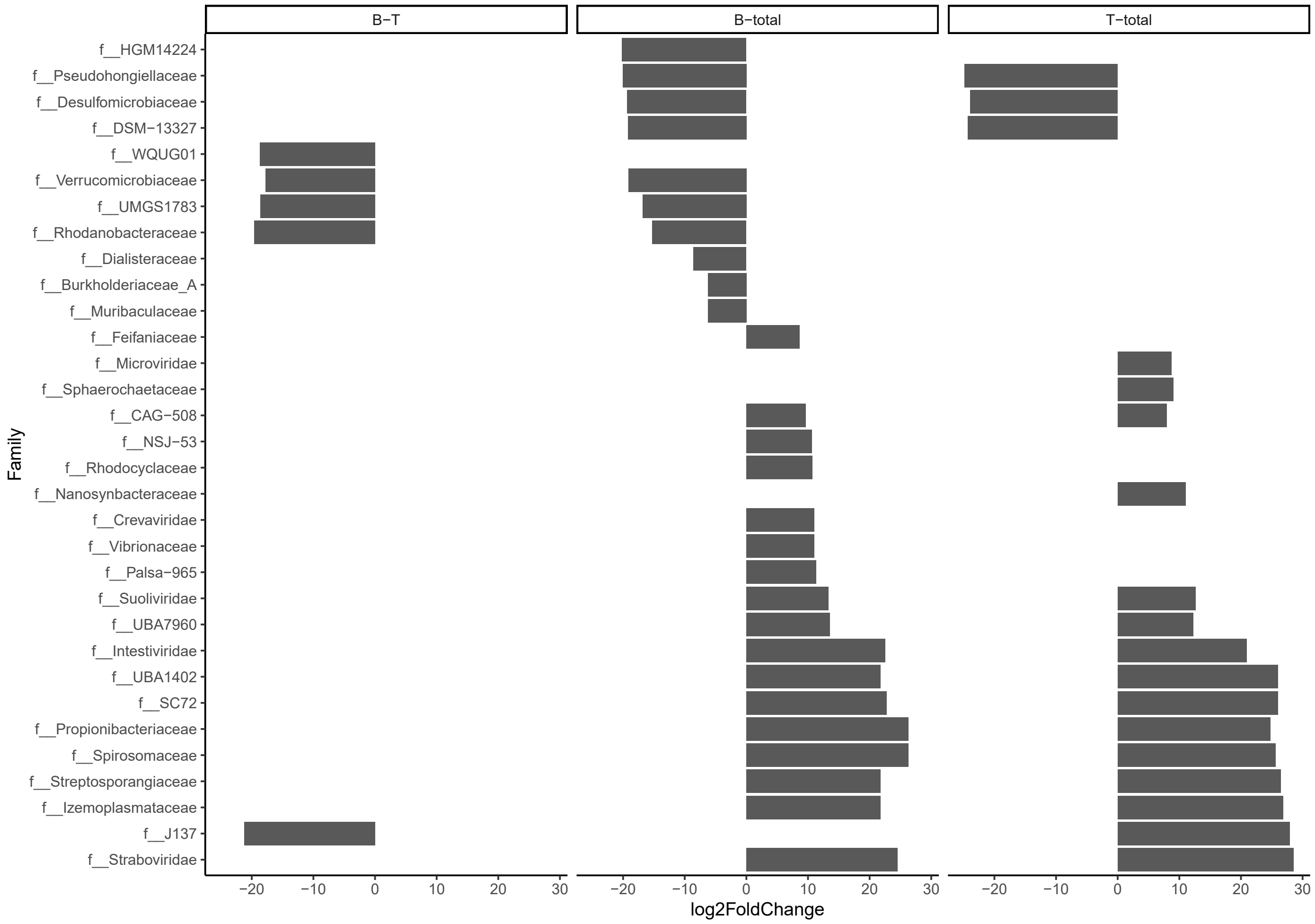

Figure S2. Bacterial families identified as differentially abundant faecal VLP fractions and total faecal community. Illumina read counts were GLM-fitted in DESeq2. Wald test was performed between the following conditions: bottom (B) VLP and top (T) VLP fractions, B-VLP and total community, T-VLP and total community. Taxa were considered significantly changed with an FDR threshold of 0.05. Effect sizes, Wald z statistic values and confidence intervals are provided in the accompanying Supplementary Data File 3.

Figure S3

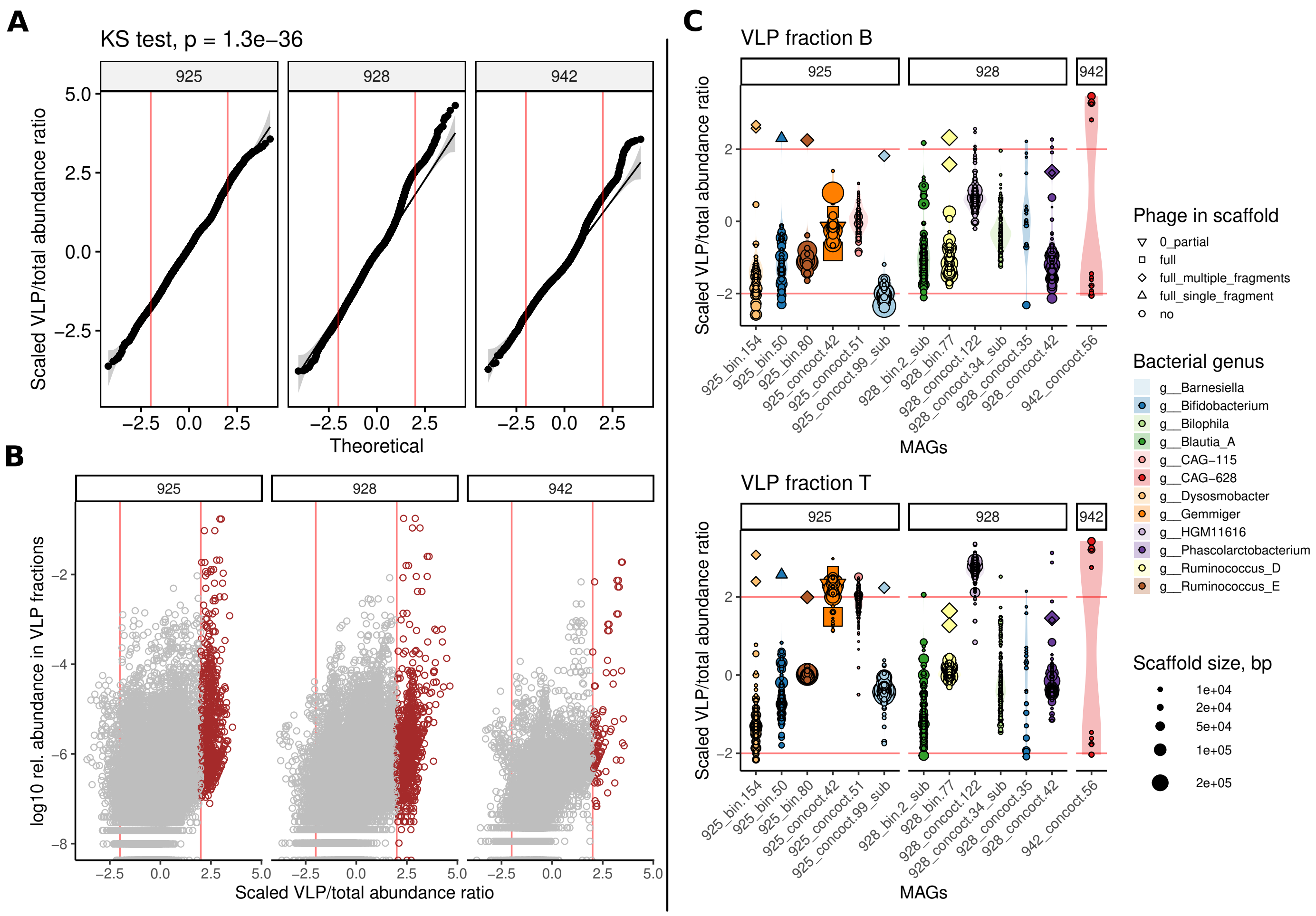

Figure S3. Selection and characteristics of bacterial MAGs enriched in the VLP fractions. A, relative abundance of genomic scaffolds in the VLP fractions versus the total community fraction deviates from the expected normal distribution (Kolmogorov-Smirnov test); **B,** an arbitrary cut-off of +2 standard deviation (SD) applied to select for genomic scaffolds overrepresented in the VLP fractions; **C,** MAGs containing such overrepresented genomic scaffolds in the top (T) and bottom (B) VLP fractions, dots correspond to individual scaffolds within the MAGs, shaped according to presence (and completeness) of phage sequence in the scaffold, dot colour corresponds to MAG taxonomy. In contrast, the size reflects the scaffold length in base pairs (bp).

Figure S4

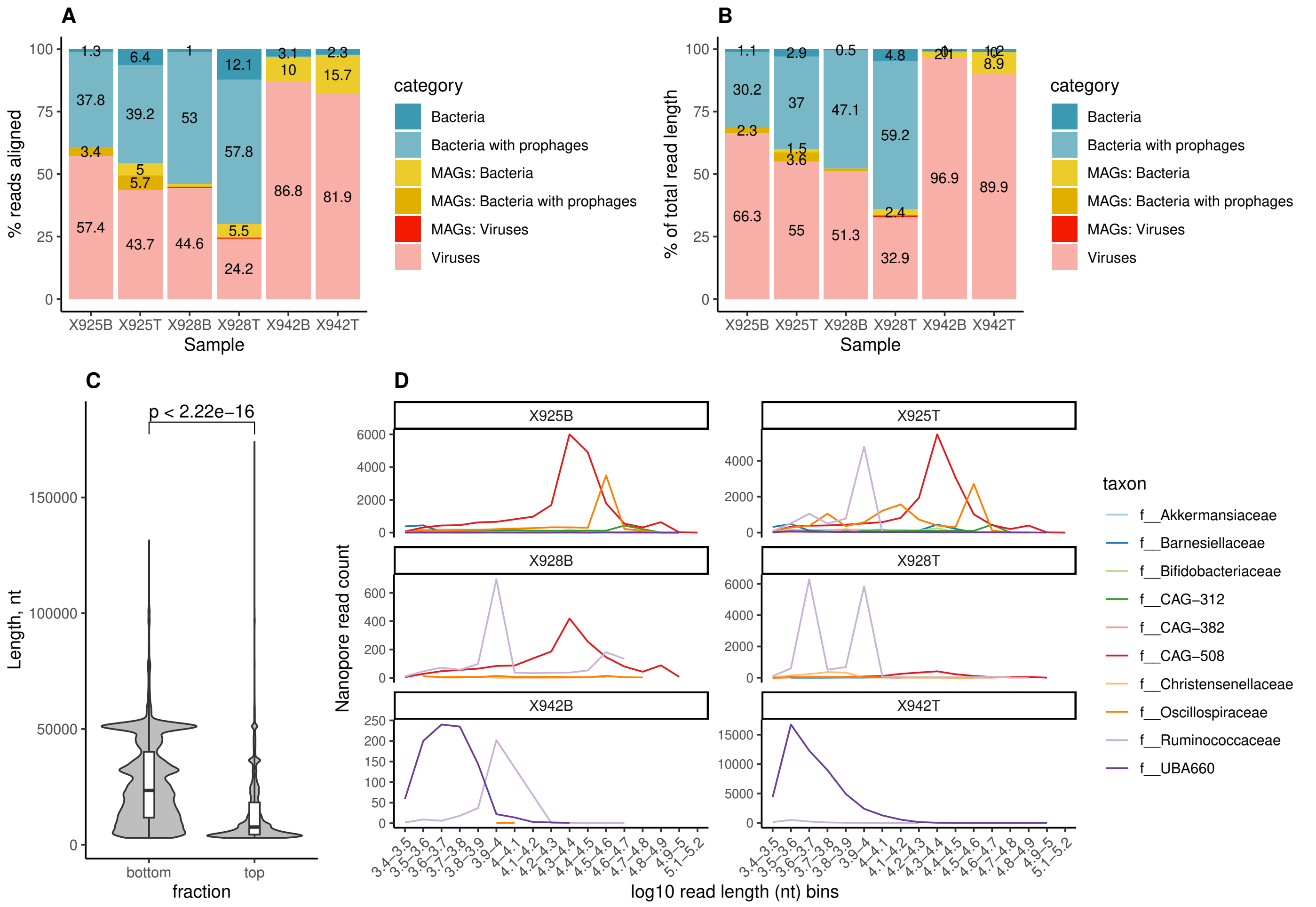

Figure S4. Composition of long nanopore sequence reads in the top (T) and bottom (B) faecal VLP fractions. A and **B**, percentage of reads or of total read length aligned to different categories of genomic scaffolds assembled from faecal donors 925, 928 and 942; **C**, difference in length of nanopore reads in VLP fractions B and T (Wilcoxon signed rank test, W = 6.84e+10); D, bacterial families demonstrating significant non-linear abundance trends along the read length gradient (bin widths of 0.1 log10 scale), Natural spline-transformed read length (df = 3) was modelled using a negative binomial GLM in edgeR, and significance was assessed with the quasi-likelihood F-test (*F*(3, 105)). Only top 10 of the most significantly differential taxa are shown with FDR < 10^{-5}). Effect sizes (spline coefficients) and *F* statistic values are provided in the accompanying Supplementary Data File 4.

Figure S5

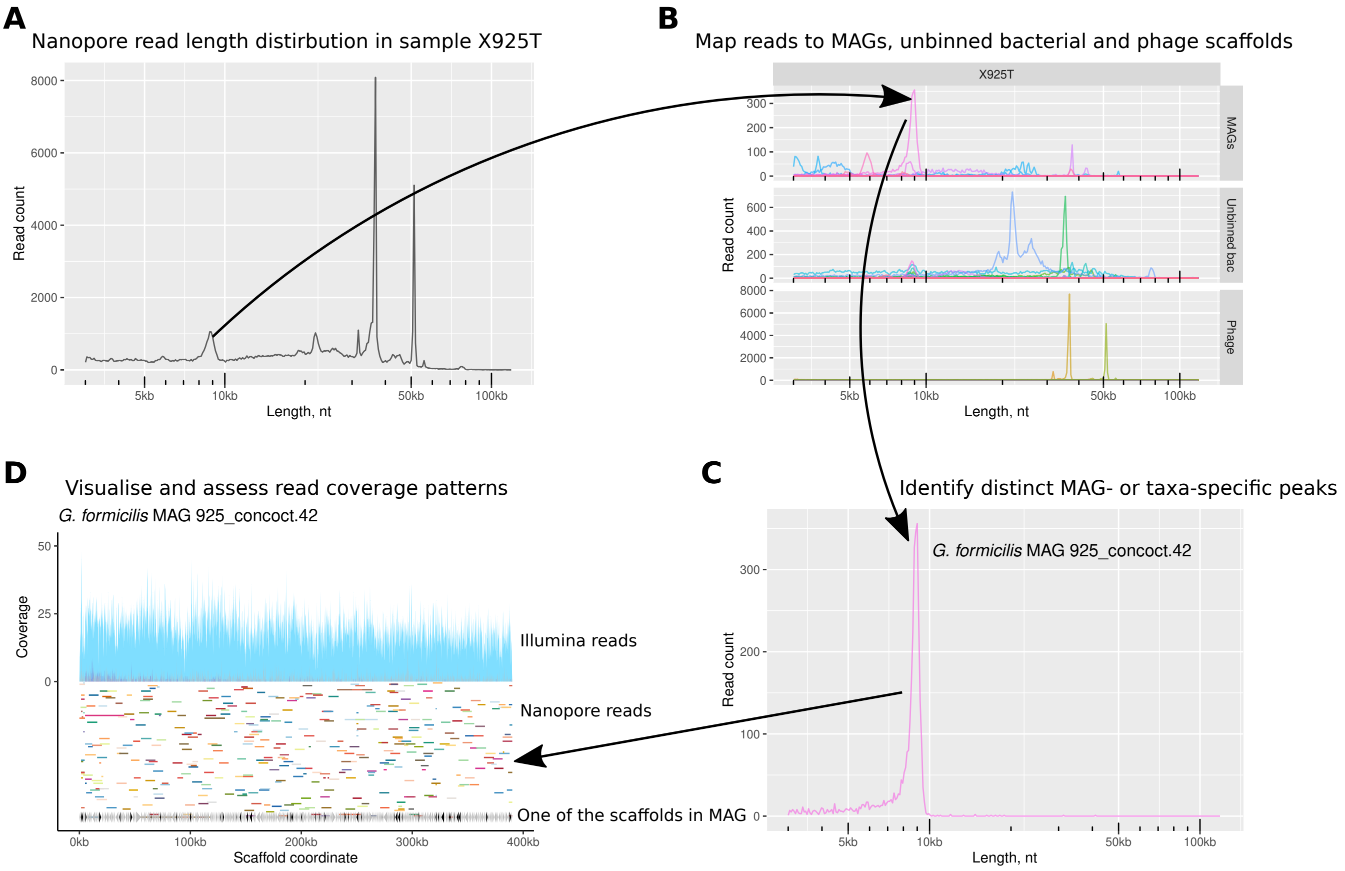

Figure S5. Manual identification of nanopore read length peak. A, read length distribution visualised for each sample (excluding reads under 1 kb); **B**, all reads are mapped to MAGs, unbinned bacterial and phage scaffolds (assembled using both Nanopore and Illumina reads). Colours represent distinct taxa; **C** and **D**, distinct MAG- or taxa-specific peaks are identified, corresponding reads selected, and their mapping to the scaffolds visualised alongside Illumina read coverage (area plot at the top); Coverage patterns are assessed manually.

Figure S6

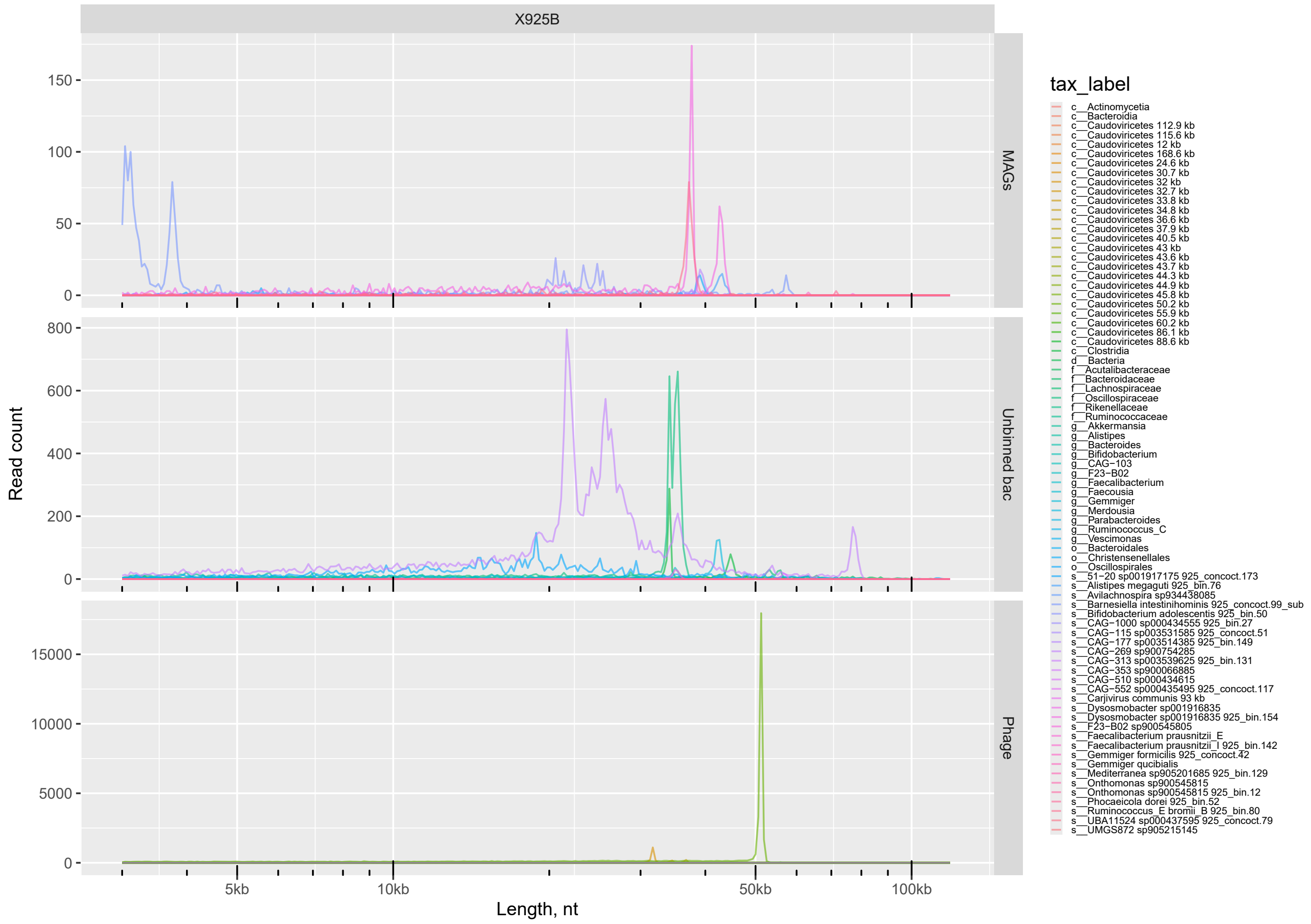

Figures S6. Length distribution of full-sized nanopore reads produced from DNA extracted from the bottom (-B) VLP fraction from sample 925. Each curve is a separate MAG (top panel)/bacterial species (middle panel)/phage genome (bottom panel); colours represent individual taxa.

Figure S7

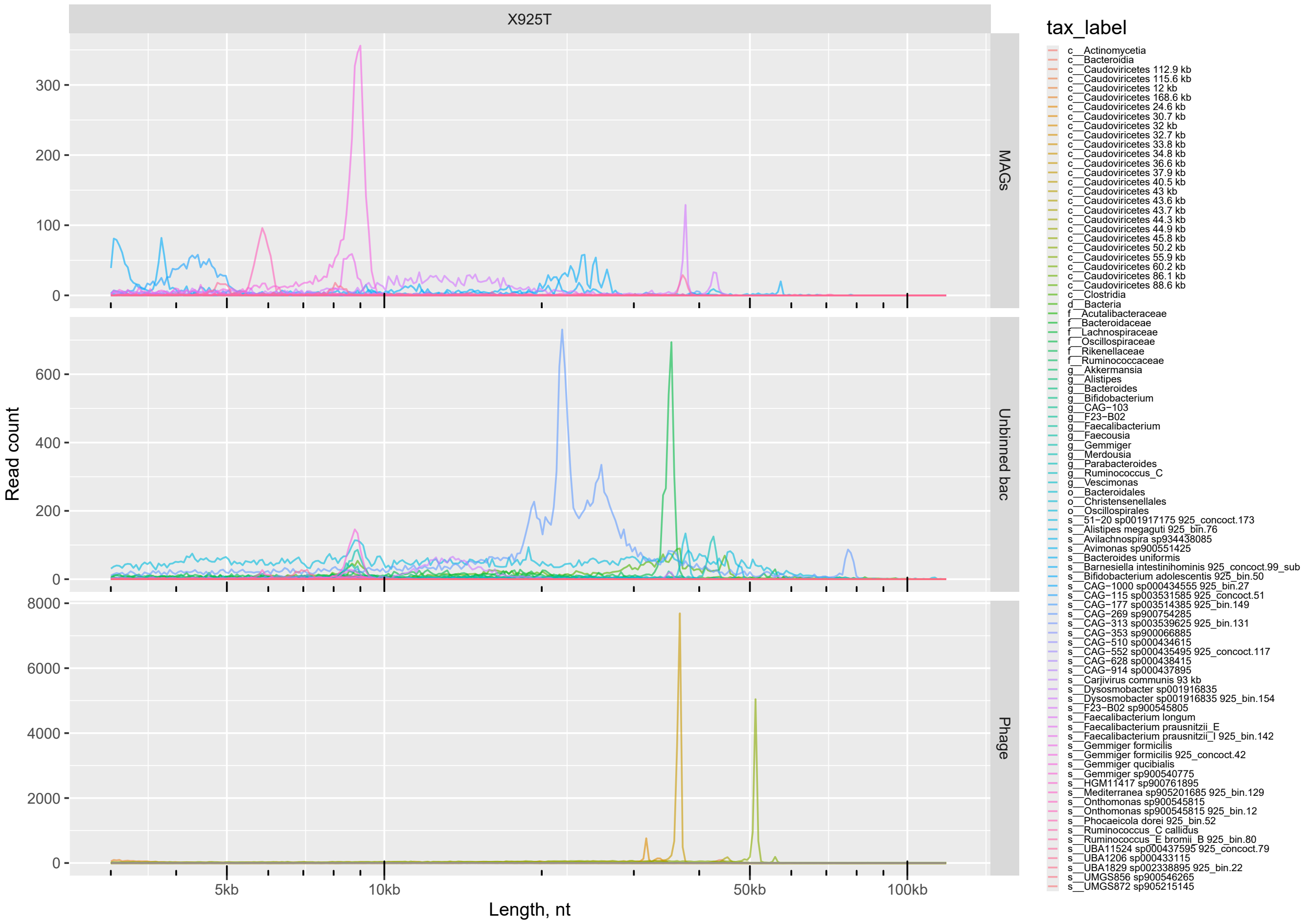

Figures S7. Length distribution of full-sized nanopore reads produced from DNA extracted from the top (-T) VLP fraction from sample 925. Each curve is a separate MAG (top panel)/bacterial species (middle panel)/phage genome (bottom panel); colours represent individual taxa.

Figure S8

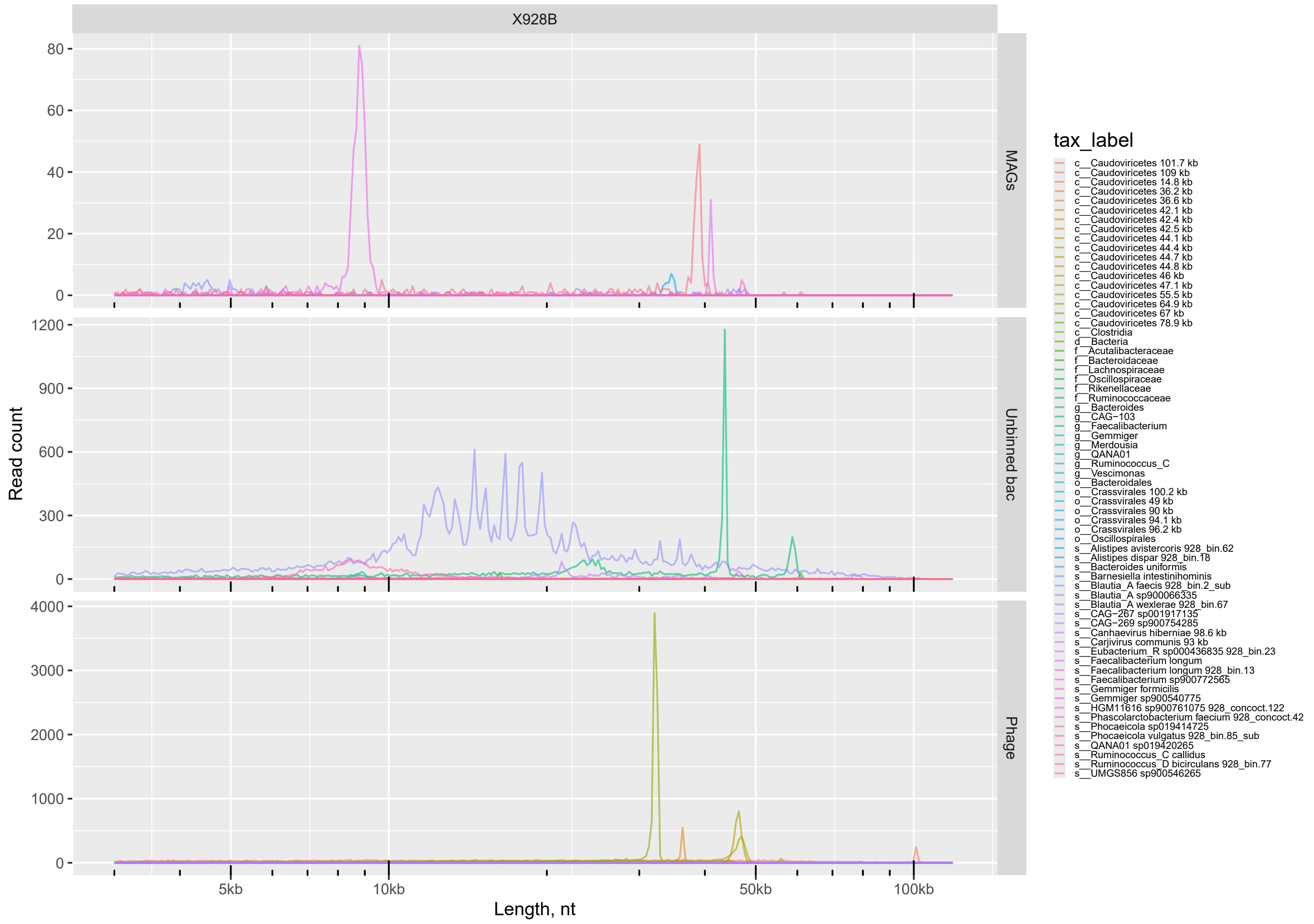

Figures S8. Length distribution of full-sized nanopore reads produced from DNA extracted from the bottom (-B) VLP fraction from sample 928. Each curve is a separate MAG (top panel)/bacterial species (middle panel)/phage genome (bottom panel); colours represent individual taxa.

Figure S9

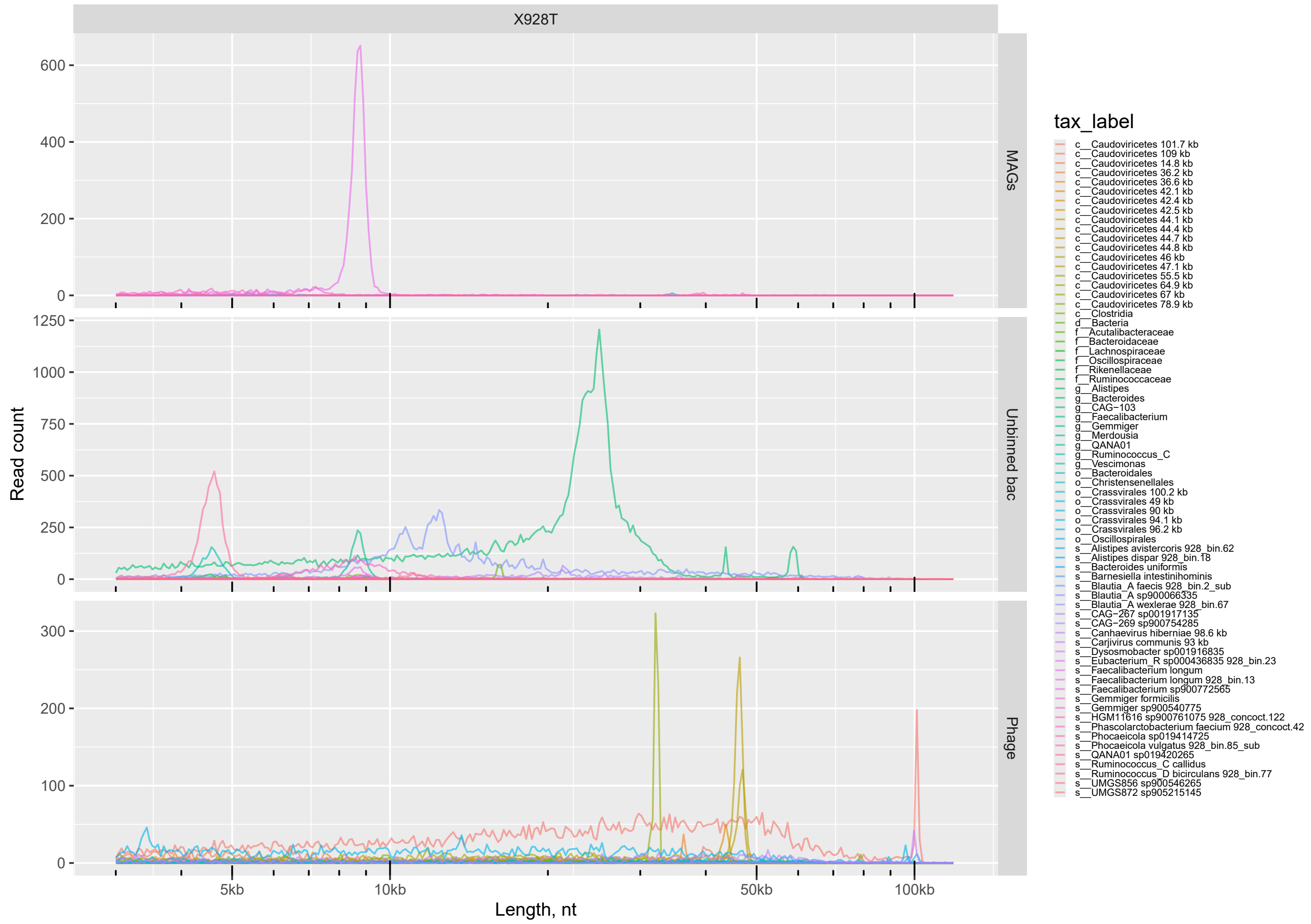

Figures S9. Length distribution of full-sized nanopore reads produced from DNA extracted from the top (-T) VLP fraction from sample 928. Each curve is a separate MAG (top panel)/bacterial species (middle panel)/phage genome (bottom panel); colours represent individual taxa.

Figure S10

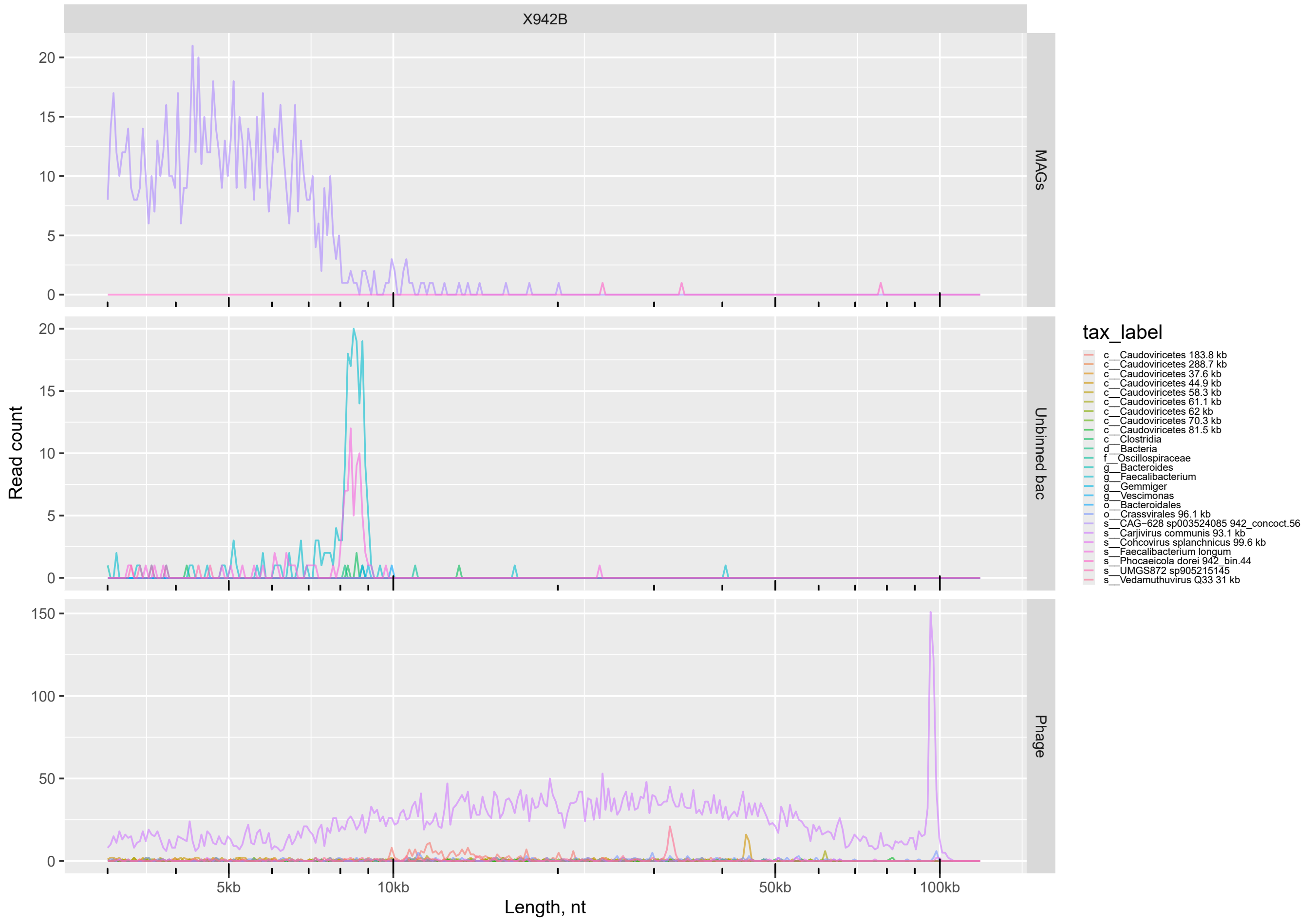

Figures S10. Length distribution of full-sized nanopore reads produced from DNA extracted from the bottom (-B) VLP fraction from sample 942. Each curve is a separate MAG (top panel)/bacterial species (middle panel)/phage genome (bottom panel); colours represent individual taxa.

Figure S11

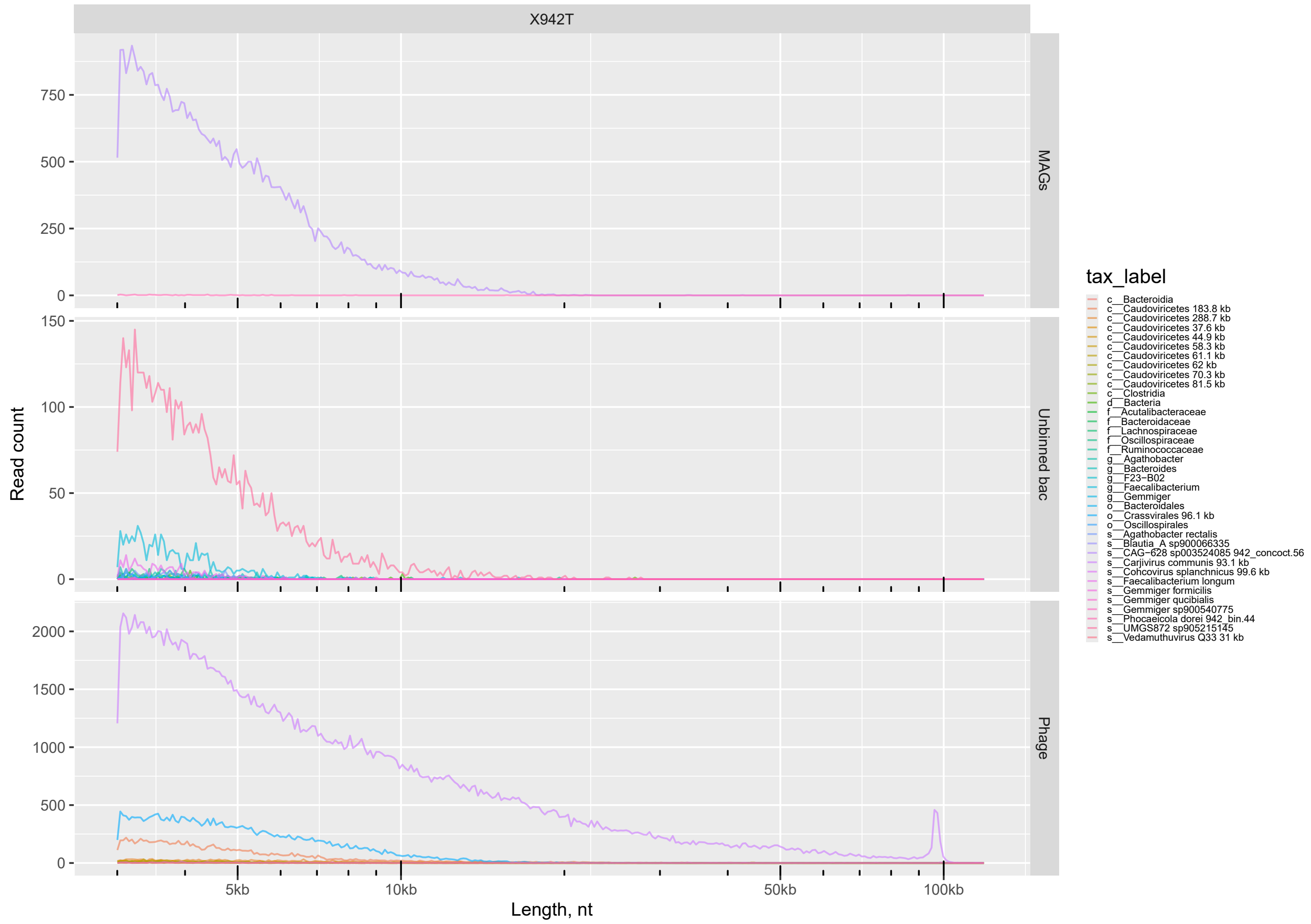

Figures S11. Length distribution of full-sized nanopore reads produced from DNA extracted from the top (-T) VLP fraction from sample 942. Each curve is a separate MAG (top panel)/bacterial species (middle panel)/phage genome (bottom panel); colours represent individual taxa.

Figure S12

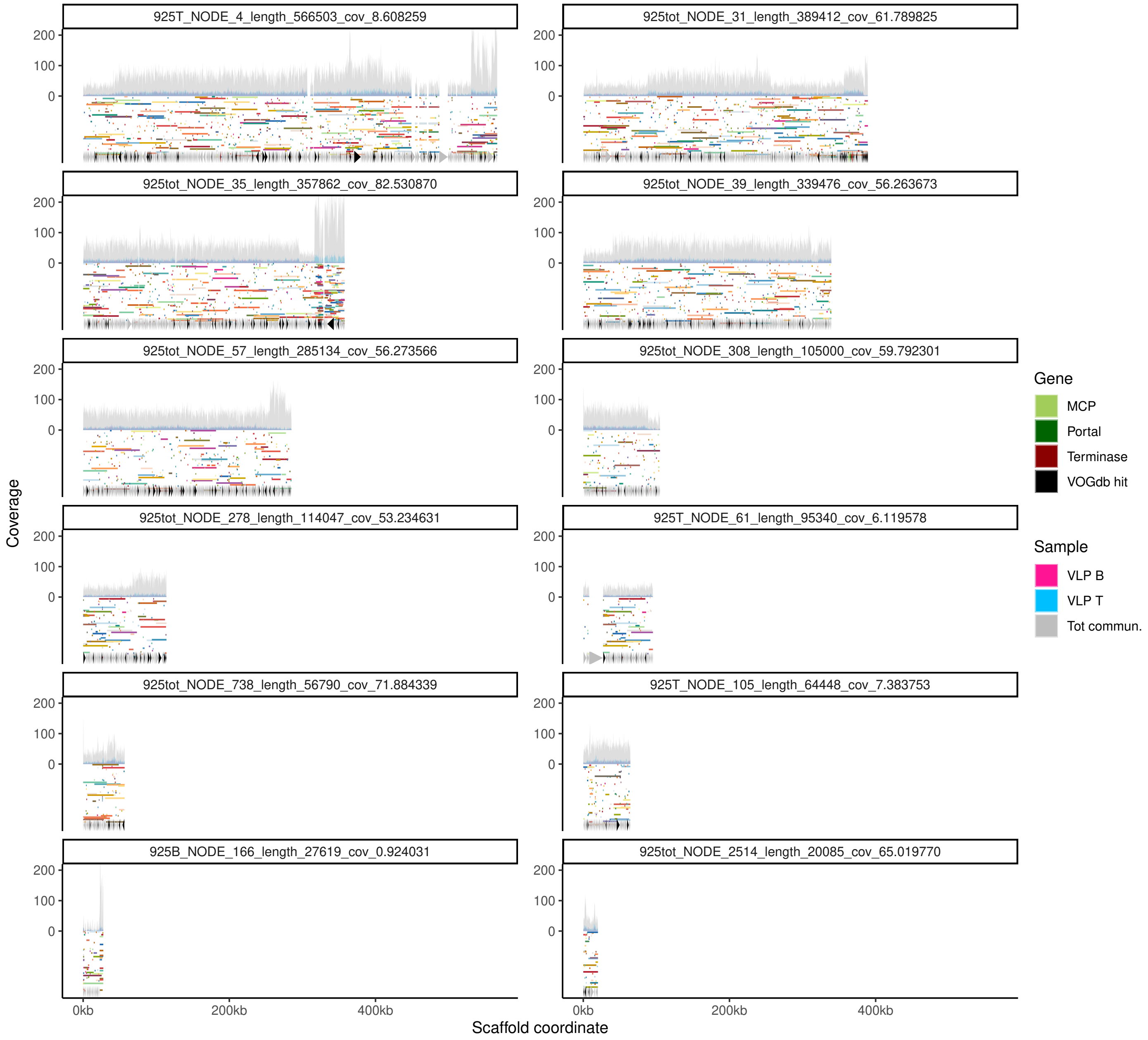

Figure S12. Read coverage of un-binned genomic scaffolds from an uncultured species F23-B02 sp900545805 from the faecal donor 925. Area plots represent coverage by short Illumina reads (DNA from VLP fractions B and T, as well as the total community DNA), whereas segments of random colours underneath that are full-sized Nanopore reads.

Figure S13

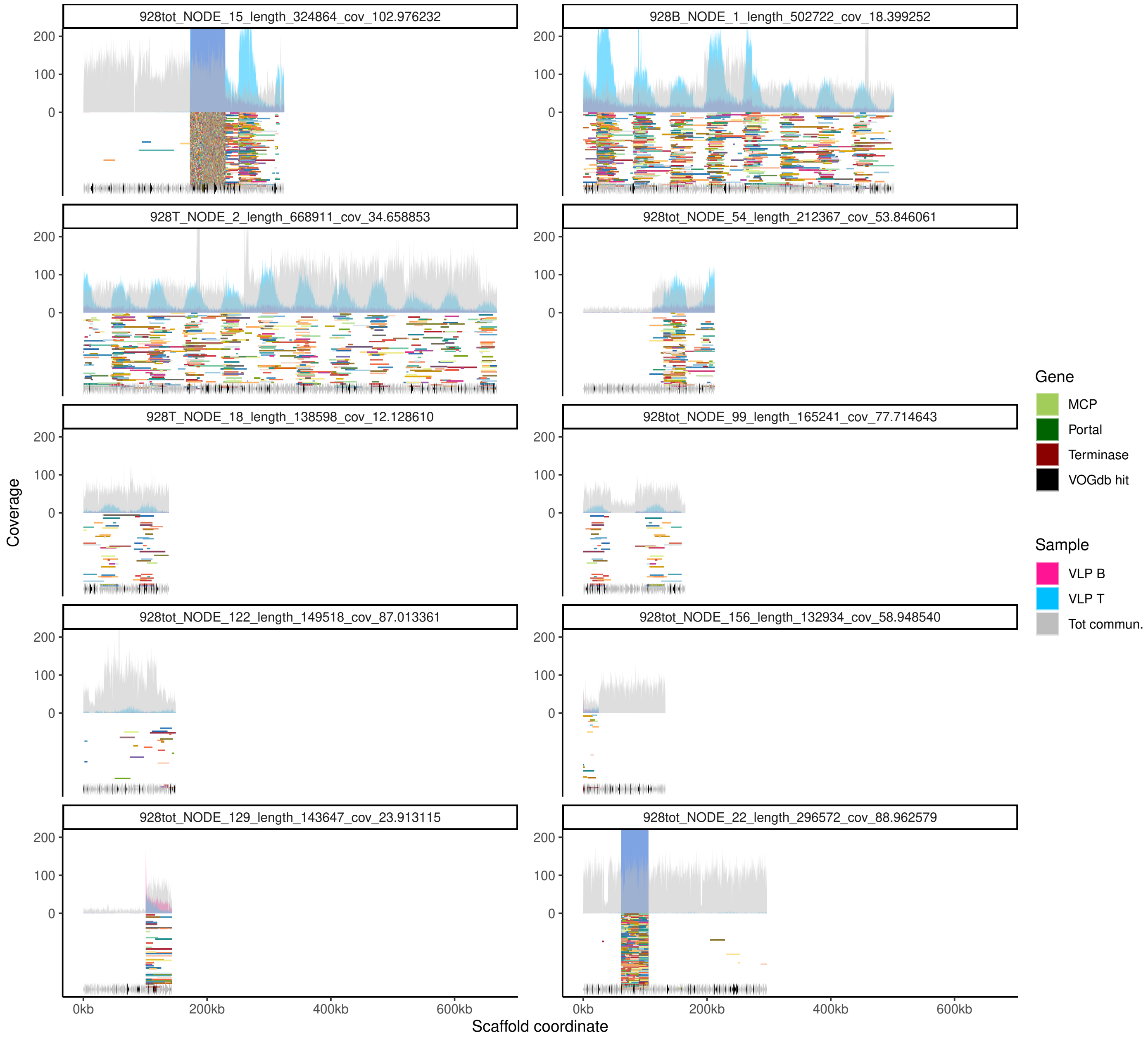

Figure S13. Read coverage of selected *Bacteroides sp.* genomic scaffolds in the faecal donor 928. Area plots represent coverage by short Illumina reads (DNA from VLP fractions B and T, as well as the total community DNA), whereas segments of random colours underneath that are full-sized Nanopore reads.
