# Supplementary material for: Large-scale capsid-mediated mobilisation of bacterial genomic DNA in the gut microbiome
Source: Nat Commun. 2026 Jan 27;17:2046. doi: 10.1038/s41467-026-68726-4 (PMC12946183; doi:10.1038/s41467-026-68726-4)
Supplement: Supplementary file 2 — Description of Additional Supplementary Files [file 41467_2026_68726_MOESM2_ESM.pdf]

Description of Additional Supplementary Files

Supplementary Data 1. Packaging events involving bacterial genomic DNA across the three human donors.

Supplementary Data 2. MS analysis of proteins in a CsCl-purified VLP fraction obtained after spontaneous lysis of a stationary phase *F. prausnitzii* ATCC27766

Supplementary Data 3. Statistical model information to accompany Figure S2.

Supplementary Data 4. Statistical model information to accompany Figure S4.
