# Supplementary material for: Large-scale capsid-mediated mobilisation of bacterial genomic DNA in the gut microbiome
Source: Nat Commun. 2026 Jan 27;17:2046. doi: 10.1038/s41467-026-68726-4 (PMC12946183; doi:10.1038/s41467-026-68726-4)
Supplement: Supplementary file 7 — Reporting Summary [file 41467_2026_68726_MOESM7_ESM.pdf]

Corresponding author(s): Andrey N. Shkoporov

Last updated by author(s): Dec 9, 2024

Reporting Summary

Nature Portfolio wishes to improve the reproducibility of the work that we publish. This form provides structure for consistency and transparency in reporting. For further information on Nature Portfolio policies, see our Editorial Policies and the Editorial Policy Checklist.

Statistics

For all statistical analyses, confirm that the following items are present in the figure legend, table legend, main text, or Methods section.

n/a	Confirmed	
☐	☒	The exact sample size (*n*) for each experimental group/condition, given as a discrete number and unit of measurement
☒	☐	A statement on whether measurements were taken from distinct samples or whether the same sample was measured repeatedly
☐	☒	The statistical test(s) used AND whether they are one- or two-sided<br>*Only common tests should be described solely by name; describe more complex techniques in the Methods section.*
☒	☐	A description of all covariates tested
☐	☒	A description of any assumptions or corrections, such as tests of normality and adjustment for multiple comparisons
☐	☒	A full description of the statistical parameters including central tendency (e.g. means) or other basic estimates (e.g. regression coefficient) AND variation (e.g. standard deviation) or associated estimates of uncertainty (e.g. confidence intervals)
☐	☒	For null hypothesis testing, the test statistic (e.g. *F*, *t*, *r*) with confidence intervals, effect sizes, degrees of freedom and *P* value noted<br>*Give P values as exact values whenever suitable.*
☒	☐	For Bayesian analysis, information on the choice of priors and Markov chain Monte Carlo settings
☒	☐	For hierarchical and complex designs, identification of the appropriate level for tests and full reporting of outcomes
☒	☐	Estimates of effect sizes (e.g. Cohen's *d*, Pearson's *r*), indicating how they were calculated

Our web collection on statistics for biologists contains articles on many of the points above.

Software and code

Policy information about availability of computer code

Data collection	ont-guppy v6.4.6; MinKNOW v22.12.7; EMBOSS v6.6.0.0; cutadapt v2.8; TrimmomaticPE v0.39; SPAdes v3.13.1; BLASTn v.2.10.0+; Bowtie2 v2.3.5.1; Samtools v1.10; MetaBAT2 dev build 2023-03-17, MaxBin2 v2.27; CONCOCT v1.1.0; DAS Tool v1.1.6; CheckM v1.2.2; GTDB-Tk v2.3.2; MMseqs2; DRAM v1.4.6; VirSorter2 v2.2.4; CheckV v1.0.1; geNomad v 1.6.1; Minimap2 v2.17-r941; bedtools v2.27.1; Phaster server (https://phaster.ca/); Collabfold v1.5.5.5; MSFragger v4.1; Fragpipe v22; Foldseek (GitHub release 8-ef4e960)
Data analysis	R environment v4.4.0; custom R scripts: DOI 10.6084/m9.figshare.26310658; gggenes v0.5.1; ggplot2 v3.5.1; ggpubr v0.6.0<br>Supplementary dataset, which includes MAG and genomic scaffold sequences, read mapping data, a complete set of R scripts and input data needed to recreate all tables and figures in the manuscript, and a complete set of raw figures describing read length distributions and genomic scaffold mapping patterns of long Nanopore reads is available at FigShare under DOI 10.6084/m9.figshare.26310658.

For manuscripts utilizing custom algorithms or software that are central to the research but not yet described in published literature, software must be made available to editors and reviewers. We strongly encourage code deposition in a community repository (e.g. GitHub). See the Nature Portfolio guidelines for submitting code & software for further information.

Data

Policy information about availability of data

All manuscripts must include a data availability statement. This statement should provide the following information, where applicable:

- Accession codes, unique identifiers, or web links for publicly available datasets
- A description of any restrictions on data availability
- For clinical datasets or third party data, please ensure that the statement adheres to our policy

Raw sequencing data is deposited at NCBI SRA database under a BioProject accession PRJNA1135972. Supplementary dataset, which includes MAG and genomic scaffold sequences, read mapping data, a complete set of R scripts and input data needed to recreate all tables and figures in the manuscript, and a complete set of raw figures describing read length distributions and genomic scaffold mapping patterns of long Nanopore reads is available at FigShare under DOI 10.6084/m9.figshare.26310658.

Research involving human participants, their data, or biological material

Policy information about studies with human participants or human data. See also policy information about sex, gender (identity/presentation), and sexual orientation and race, ethnicity and racism.

Reporting on sex and gender	This study reports metagenomic analysis of faecal samples from only three human donors. This is not sufficient to make any conclusions about sex- or gender-specific differences in faecal virome composition and/or phage transduction activity.
Reporting on race, ethnicity, or other socially relevant groupings	This study reports metagenomic analysis of faecal samples from only three human donors. No conclusions/analyses of these parameters were included.
Population characteristics	This study reports metagenomic analysis of faecal samples from only three human donors. No conclusions/analyses of these parameters were included.
Recruitment	This study used faecal samples from previously recruited and consented faecal donors. Study protocol APC_055 (DOI 10.1016/j.chom.2019.09.009).
Ethics oversight	CREC - Clinical Research Ethics Committee, Cork, Ireland

Note that full information on the approval of the study protocol must also be provided in the manuscript.

Field-specific reporting

Please select the one below that is the best fit for your research. If you are not sure, read the appropriate sections before making your selection.

- [x] Life sciences
- [ ] Behavioural & social sciences
- [ ] Ecological, evolutionary & environmental sciences

For a reference copy of the document with all sections, see nature.com/documents/nr-reporting-summary-flat.pdf

Life sciences study design

All studies must disclose on these points even when the disclosure is negative.

Sample size	This is a small-scale proof of concept study. This study reports metagenomic analysis of faecal samples from only three human donors.
Data exclusions	None
Replication	No attempts were made to replicate main findings.
Randomization	No grouping.
Blinding	No grouping.

Reporting for specific materials, systems and methods

We require information from authors about some types of materials, experimental systems and methods used in many studies. Here, indicate whether each material, system or method listed is relevant to your study. If you are not sure if a list item applies to your research, read the appropriate section before selecting a response.

Materials & experimental systems

n/a	Involved in the study
☒	☐ Antibodies
☒	☐ Eukaryotic cell lines
☒	☐ Palaeontology and archaeology
☒	☐ Animals and other organisms
☒	☐ Clinical data
☒	☐ Dual use research of concern
☒	☐ Plants

Methods

n/a	Involved in the study
☒	☐ ChIP-seq
☒	☐ Flow cytometry
☒	☐ MRI-based neuroimaging

Plants

Seed stocks	Work does not involve plants.
Novel plant genotypes	Work does not involve plants.
Authentication	Work does not involve plants.
